# Effects of 12-Week Multicomponent Training Program on Body Composition, Metabolic Health, and Physical Performance in Middle-Aged and Older Women: Exploratory Role of Baseline Adiposity

**DOI:** 10.3390/sports14050204

**Published:** 2026-05-15

**Authors:** Citlali Campos-Hernández, Tatiana Romero-García, Héctor Frayde-Gómez, Cristhian Emmanuel López-Campos, María Jossé Navarro-Ibarra, Juan Carlos Borbón-Román, Juan Pablo Machado-Parra, Victor Enrique Porras-Alvarado, Mario Israel Oregel-Cortez

**Affiliations:** 1 Facultad de Deportes, Universidad Autónoma de Baja California, Mexicali 21289, Baja California, Mexico; citlali.campos@uabc.edu.mx (C.C.-H.); tatiana.romero@uabc.edu.mx (T.R.-G.); lopez.cristhian92@uabc.edu.mx (C.E.L.-C.); carlos.borbon@uabc.edu.mx (J.C.B.-R.); vporras@uabc.edu.mx (V.E.P.-A.); 2 Centro de Ciencias de la Salud Mexicali, Universidad Autónoma de Baja California, Mexicali 21040, Baja California, Mexico; fraydeh@uabc.edu.mx; 3 Hospital Regional de Especialidad No. 30, Instituto Mexicano del Seguro Social, Mexicali 21100, Baja California, Mexico; 4 Facultad de Medicina Mexicali, Universidad Autónoma de Baja California, Mexicali 21000, Baja California, Mexico; maria.navarro.ibarra@uabc.edu.mx; 5 Facultad de Deportes, Universidad Autónoma de Baja California, Ensenada 22890, Baja California, Mexico; machado.juan@uabc.edu.mx

**Keywords:** high-intensity interval training, functional training, body fat percentage, aerobic capacity, metabolic health, middle-aged and older women, exercise intervention, functional performance

## Abstract

Combined functional training (FT), high-intensity interval training (HIIT) and aquatic exercise may improve health-related fitness in aging populations; however, the influence of baseline adiposity on training responses remains unclear. This study evaluated the effects of a 12-week multicomponent training program on aerobic capacity, body composition, metabolic health, and physical performance in middle-aged and older women and explored whether baseline body fat percentage modulated these responses. Thirty-four women (50–72 years) were assigned to a control group (Ctrl, *n* = 10) or an exercise group, stratified into normal fat (NF%, *n* = 10) and high fat (HF%, *n* = 14). The intervention included three weekly 60 min sessions consisting of HIIT, FT, and aquatic-based interval and resistance exercises, while controls maintained their habitual lifestyle without structured exercise. Significant improvements were observed in VO_2_max, skeletal muscle mass, fasting insulin, triglycerides, total cholesterol, HDL cholesterol, and functional performance. Baseline adiposity influenced metabolic adaptations, with greater improvements in the HF% group. These findings suggest that multicomponent training may improve cardiometabolic health and physical performance; however, the results should be interpreted cautiously due to the quasi-experimental design and small sample size.

## 1. Introduction

Aging is associated with diverse physiological adjustments that negatively affect body composition and metabolism. Among these changes, the progressive loss of muscle mass, increased adipose tissue accumulation, reduced insulin sensitivity, and deterioration of lipid profiles are particularly notable. Together, these metabolic disturbances increase the likelihood of developing chronic diseases such as dyslipidemia, metabolic syndrome, and type 2 diabetes mellitus. These conditions are of particular concern because they not only affect physical health but also compromise functional capacity and quality of life in middle-aged and older women. Due to these age-related physiological adaptations, identifying effective strategies to preserve metabolic health and physical function in middle-aged and older women has become an important public health priority. In this context, regular physical activity is recognized as one of the most effective strategies to mitigate the adverse effects of aging on body composition and metabolic health [1,2].

In recent years, high-intensity interval training (HIIT) and functional training (FT) have emerged as promising alternatives to traditional exercise programs in older adults [3,4,5]. HIIT is characterized by short bouts of vigorous exercise interspersed with recovery periods, while FT focuses on multi-joint movements that simulate activities of daily living, promoting strength, coordination, and functional independence [6]. Notably, both training modalities can be performed in relatively short sessions, which may enhance adherence in older populations, where fatigue, physical limitations, and time constraints are common barriers to regular physical activity.

In addition, aquatic-based exercise has gained attention as a complementary training modality in older adults, as it allows for the performance of aerobic and resistance exercises with reduced joint loading while maintaining sufficient physiological stimulus. This may facilitate adherence and expand training possibilities in populations with musculoskeletal limitations or reduced exercise tolerance.

Despite the increasing popularity of HIIT and FT, current scientific evidence regarding the effects of these training strategies in middle-aged and older populations remains limited. Most studies have focused on young or middle-aged individuals, and many investigations have evaluated these training modalities independently. Consequently, relatively few studies have examined the effects of programs that combine FT and HIIT in middle-aged and older women. This issue is especially relevant because physiological changes associated with aging and the menopausal transition can significantly influence fat distribution, insulin sensitivity, and overall cardiometabolic risk [7,8].

Previous studies have shown that training programs based on FT or HIIT may improve several health-related outcomes, including increases in maximal oxygen consumption, improvements in muscular strength and endurance, and reductions in body fat. These adaptations suggest that both training modalities may contribute to improvements in cardiorespiratory fitness, body composition, and musculoskeletal health [9]. However, limited evidence exists regarding the effects of training programs that integrate both FT and HIIT in middle-aged and older women and their simultaneous impact on metabolic health indicators, body composition, and physical fitness.

Additionally, baseline adiposity may influence the magnitude of training-induced adaptations, as individuals with higher body fat levels often present greater metabolic dysregulation and may respond differently to exercise interventions. Understanding this variability is important for optimizing exercise prescription and improving cardiometabolic outcomes in this population.

To our knowledge, few studies have simultaneously examined the combined effects of functional training, high-intensity interval training, and aquatic exercise while also exploring baseline adiposity as a potential modulator of training responsiveness in middle-aged and older women.

Therefore, the aim of the present study was to evaluate the effects of a multicomponent training program including FT, HIIT, and aquatic exercise on body composition, metabolic health, and aerobic capacity in middle-aged and older women compared with a control group that maintained their habitual lifestyle, defined as continuing their normal daily activities without engaging in any structured or supervised exercise program during the same intervention period. Additionally, this study explored whether baseline adiposity levels could influence the magnitude of the physiological adaptations induced by the training program. It was hypothesized that participation in the combined FT + HIIT program would lead to significant improvements in key indicators of metabolic health, body composition, and physical fitness, thereby contributing to healthier and more active aging.

## 2. Materials and Methods

### 2.1. Ethical Approval Statement

This study was approved by the Ethics and Research Committee of the Faculty of Sports at the Autonomous University of Baja California (Approval No. 515/2024-1). All participants were fully informed about the study procedures, potential risks, and benefits and provided written informed consent prior to participation.

### 2.2. Study Design

This study followed a quasi-experimental design with pre–post measurements conducted over a 12-week intervention period. The primary aim was to evaluate the effects of a combined FT and HIIT program on body composition, metabolic markers, and physical fitness in middle-aged and older women and to explore the potential influence of baseline adiposity on the magnitude of these adaptations.

Participants were allocated to either a control group or an exercise intervention group based on availability and willingness to participate in the training sessions, rather than through randomization. The intervention group was subsequently stratified according to baseline body fat percentage into normal fat (NF%) and high fat (HF%) subgroups for exploratory analyses.

All participants underwent baseline assessments (week 0) and post-intervention evaluations (week 12) under standardized conditions.

### 2.3. Participants

**Figure 1:**
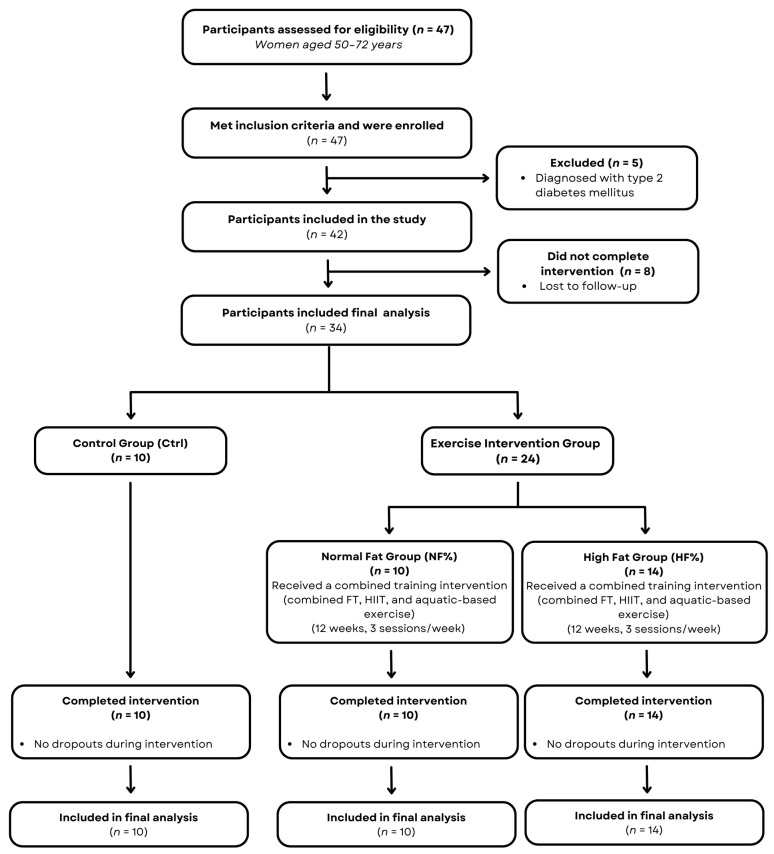
Flow diagram of participant recruitment, allocation, follow-up, and analysis. Ctrl: control group; NF%: normal fat group; HF%: high fat group; FT: functional training; HIIT: high-intensity interval training.

A total of 47 women were initially recruited for this study. Five participants were excluded due to a diagnosis of type 2 diabetes mellitus, and eight did not complete the 12-week intervention. Therefore, 34 participants were included in the final analysis (Ctrl, *n* = 10; NF%, *n* = 10; HF%, *n* = 14). Figure 1 presents a flow diagram of participant recruitment, allocation, follow-up, and analysis.

All participants were women aged between 50 and 72 years and were classified as physically inactive based on self-reported physical activity levels.

Participants were allocated to either a control group (Ctrl, *n* = 10) or an exercise intervention group (*n* = 24) based on their availability and willingness to participate in the training sessions. The intervention group was further stratified according to baseline body fat percentage into NF%, <32% and HF%, ≥32% groups for exploratory analyses.

Inclusion criteria were: (i) female sex, (ii) age between 50 and 72 years, and (iii) absence of structured exercise participation in the previous six months. Exclusion criteria included: (i) diagnosis of uncontrolled chronic diseases, (ii) musculoskeletal limitations, and (iii) medical contraindications to exercise.

Adherence to the training program was high, with attendance rates of 96.66% in the NF% group and 96.42% in the HF% group.

All participants provided written informed consent prior to participation.

### 2.4. Exercise Intervention

The exercise intervention consisted of a 12-week combined training program integrating FT, HIIT, and aquatic-based exercise. Participants performed three supervised sessions per week on non-consecutive days, with each session lasting approximately 60 min.

Each week included three distinct training sessions: (i) one land-based HIIT session, (ii) one FT session targeting major muscle groups, and (iii) one combined session performed in an aquatic environment incorporating both FT and interval-based exercises. This structure was designed to promote complementary physiological adaptations while reducing joint stress and improving adherence.

Each session followed a standardized structure consisting of a warm-up (10 min), a main training phase (40 min), and a cool-down period (10 min). The warm-up included low-intensity aerobic activity and dynamic mobility exercises.

The HIIT sessions consisted of time-based intervals performed at intensities ≥85% of maximum heart rate (HRmax), interspersed with recovery periods. Heart rate was continuously monitored using a commercially available smartwatch (Apple Watch series 9, Apple Inc., Cupertino, CA, USA) to ensure participants reached the target intensity zones.

The FT sessions included multi-joint resistance exercises using body weight, free weights, and resistance machines, focusing on major muscle groups. Training intensity was progressively increased from approximately 60% to 75% of the estimated one-repetition maximum (1RM). The 1RM was estimated using the Brzycki equation based on submaximal repetitions, ensuring a safe and standardized assessment of maximal strength in this population [10].

The aquatic sessions combined aerobic and resistance-based movements adapted to the physical properties of water, including buoyancy and resistance, allowing for the execution of functional and interval-based exercises with reduced joint impact, with the aim of eliciting a training stimulus comparable to that of land-based sessions.

Training load, complexity, and intensity were progressively increased throughout the intervention to promote physiological adaptations while minimizing injury risk. All sessions were supervised by certified professionals, and load adjustments were individualized based on participants’ performance and tolerance. A detailed weekly structure of the training program is presented in Table 1.

### 2.5. Anthropometric Measures

Anthropometric and body composition variables were assessed using a multifrequency bioelectrical impedance analyzer (InBody 720, Biospace Co., Ltd., Seoul, Republic of Korea). The variables obtained included body mass, body fat percentage, fat mass, and skeletal muscle mass.

All assessments were performed under standardized conditions. Participants were instructed to fast for at least 8 h, avoid vigorous physical activity for 24 h prior to testing, and refrain from consuming alcohol or caffeine before the assessment. All measurements were conducted at 8:30 a.m., with participants wearing light clothing and barefoot, following the manufacturer’s guidelines.

For exploratory subgroup analyses, participants in the intervention group were stratified according to baseline body fat percentage using a threshold of 32%, a commonly applied criterion to identify elevated adiposity and increased cardiometabolic risk in adult women [11,12]. Based on this classification, participants were categorized as NF%, <32% or HF%, ≥32%.

This stratification approach was implemented to examine whether baseline adiposity modulates the magnitude of physiological and functional adaptations induced by the FT + HIIT intervention.

### 2.6. Biochemical Analyses

After an overnight fast of at least 8 h, 5 mL of peripheral venous blood was collected at 8:30 a.m. under standardized conditions. Blood samples were drawn into polypropylene tubes without anticoagulant (Vacutainer System, BD Biosciences, Franklin Lakes, NJ, USA) and centrifuged at 4000 rpm for 15 min to obtain serum.

The serum concentrations of total cholesterol, glucose, triglycerides, and high-density lipoprotein cholesterol (HDL-C) were determined using an automatic analyzer (Spin 120, SpinReact, Barcelona, Spain) following standard enzymatic colorimetric methods. Insulin levels were measured by enzyme-linked immunosorbent assay (ELISA) using commercial kits according to the manufacturer’s instructions (Insulin IN374S, Calbiochem, San Diego, CA, USA).

Glucose concentrations were expressed in mg/dL and insulin in μU/mL. Insulin resistance was estimated using the Homeostatic Model Assessment for Insulin Resistance (HOMA-IR), calculated as (fasting insulin [μU/mL] × fasting glucose [mg/dL])/405. The quantitative insulin sensitivity check index (QUICKI) was calculated as 1/[log(fasting insulin) + log(fasting glucose)]. Additionally, the triglyceride-to-HDL cholesterol ratio (TG/HDL) was calculated as a surrogate marker of cardiometabolic risk.

### 2.7. Aerobic Fitness Test

Aerobic capacity was estimated using the Rockport One-Mile Walk Test, a validated submaximal field test commonly used to assess cardiorespiratory fitness in adult and older populations [13]. Participants were instructed to walk one mile (1609 m) as fast as possible on a flat surface while maintaining a steady pace.

Heart rate was recorded immediately upon the completion of the test using a commercially available smartwatch (Apple Watch series 9, Apple Inc., Cupertino, CA, USA) [14]. Time to complete the distance was also recorded. Maximal oxygen uptake (VO_2_max) was estimated using validated predictive equations based on age, body weight, walking time, and heart rate.

All tests were conducted under standardized conditions at the same time of day (8:30 a.m.) and were supervised by trained personnel to ensure consistency and reliability.

### 2.8. Functional Performance Assessment

Physical performance was evaluated using a functional test battery adapted from the McCloy system [15], which has been widely used to assess muscular endurance and functional capacity in adult populations. The assessment included six exercises: squats, push-ups, sit-ups, jumping jacks, mountain climbers, and burpees.

Each exercise was performed for 60 s, and the maximum number of repetitions completed within this time was recorded. Participants were instructed to perform each movement with proper technique and at a self-selected maximal effort. Standardized instructions and demonstrations were provided prior to testing to ensure consistency across participants.

All tests were conducted under standardized conditions and supervised by trained personnel. The same evaluators conducted both pre- and post-intervention assessments to minimize inter-observer variability. The number of repetitions achieved in each exercise was used as an indicator of muscular endurance and overall functional performance.

### 2.9. Statistical Analysis

Data are presented as the mean ± standard error of the mean (SEM). The normality of data distribution was assessed using the Shapiro–Wilk test.

Within-group comparisons between pre- and post-intervention values were performed using paired *t*-tests for normally distributed variables and Wilcoxon signed-rank tests for non-normally distributed variables.

Between-group differences in change scores (Δ) were analyzed according to the number of groups compared. For comparisons between two groups (control vs. intervention), independent sample *t*-tests were used for normally distributed variables and Mann–Whitney U tests for non-parametric data. For comparisons among three groups (Ctrl, NF%, and HF%), a one-way analysis of variance (ANOVA) was used for normally distributed variables and Kruskal–Wallis tests for non-parametric data.

When significant differences were detected in the three-group comparisons, post hoc analyses were conducted using multiple comparison tests. Dunnett’s test was applied for comparisons against the control group, while Tukey’s test was used to evaluate all pairwise comparisons in parametric data. For non-parametric data, Dunn’s test was used. Only statistically significant post hoc comparisons are reported in the tables, presented using superscript notation (a: *p* < 0.05, b: *p* < 0.01, c: *p* < 0.001 vs. control group).

Effect sizes for two-group comparisons were calculated using Cohen’s d based on change scores (Δ) and interpreted according to conventional thresholds as trivial (<0.2), small (0.2–0.49), moderate (0.5–0.79), and large (≥0.8). Statistical significance was set at *p* < 0.05.

Given the quasi-experimental design and the absence of covariance adjustments for baseline differences between groups, the results should be interpreted with caution.

All statistical analyses were performed using GraphPad Prism software version 9.5.0 (GraphPad Software, San Diego, CA, USA).

## 3. Results

No significant baseline differences were observed between the control and intervention groups for any measured variable (*p* > 0.05; Table 2).

### 3.1. Changes in Aerobic Capacity, Body Composition, and Metabolic Parameters

Table 3 summarizes the changes in aerobic capacity, body composition, and metabolic biomarkers following the 12-week intervention.

A significant between-group difference in change scores was observed for VO_2_max (*p* = 0.035), indicating a greater improvement in aerobic capacity in the intervention group compared with the control group. Specifically, VO_2_max increased from 12.55 ± 1.16 to 15.71 ± 1.06 mL·kg^−1^·min^−1^ in the intervention group, whereas only minimal changes were observed in the control group (14.15 ± 2.33 to 14.36 ± 2.23 mL·kg^−1^·min^−1^). The magnitude of this effect was classified as large (Cohen’s d = 1.10).

Regarding body composition, a significant increase in skeletal muscle mass was observed in the intervention group compared with the control group (*p* = 0.038), corresponding to a large effect size (d = 0.81). In contrast, no significant between-group differences were detected for body fat percentage or body mass index (BMI) (*p* = 0.850 and *p* = 0.410, respectively).

In terms of glucose metabolism, fasting insulin levels decreased significantly in the intervention group compared with the control group (*p* = 0.030), with a moderate effect size (d = −0.79). Although HOMA-IR showed a reduction following the intervention, the between-group difference did not reach statistical significance (*p* = 0.094). No significant changes were observed in fasting glucose levels (*p* = 0.830).

Significant improvements were also observed in lipid profile parameters. Triglyceride levels decreased in the intervention group relative to the control group (*p* = 0.029; d = −0.66), while total cholesterol was significantly reduced (*p* = 0.014). Additionally, HDL cholesterol increased significantly following the intervention (*p* < 0.001), with a large effect size (d = 1.53).

Functional performance outcomes assessed by the McCloy physical fitness test are presented in Table 4. Participants in the intervention group demonstrated consistent improvements across most exercises compared with the control group.

Significant increases were observed in squats (*p* = 0.008; d = 1.06), sit-ups (*p* = 0.005; d = 1.12), jumping jacks (*p* = 0.006; d = 0.98), mountain climbers (*p* = 0.005; d = 1.13), and burpees (*p* = 0.011; d = 2.85), indicating large improvements in muscular endurance and functional capacity. Although the increase in push-ups did not reach statistical significance (*p* = 0.058), a large effect size was observed (d = 1.00), suggesting a meaningful improvement in upper-body muscular endurance.

### 3.2. Influence of Baseline Adiposity on Training-Induced Adaptations

To further examine whether baseline adiposity influenced the magnitude of training-induced adaptations, participants were stratified into three groups: control (Ctrl), normal NF%, and HF%.

Comparisons among groups are presented in Table 5. Significant between-group differences in change scores were observed for VO_2_max, fasting insulin, and HDL cholesterol (*p* < 0.05). Post hoc analyses, using the control group as the reference, indicated that the HF% group exhibited greater improvements in VO_2_max and fasting insulin compared with Ctrl. In contrast, HDL cholesterol levels were significantly higher in both the NF% and HF% groups compared with Ctrl, indicating a consistent improvement in lipid profile regardless of baseline adiposity. Superscript letters denote the level of statistical significance compared with the control group (a *p* < 0.05, b *p* < 0.01, c *p* < 0.001).

No statistically significant differences were observed among groups for body composition variables, including body fat percentage and body mass index (BMI) (*p* > 0.05), suggesting that body composition adaptations were relatively similar across groups.

Functional performance outcomes are presented in Table 6. Significant between-group differences in change scores were detected for push-ups, sit-ups, jumping jacks, mountain climbers, and burpees (*p* < 0.05). However, although a significant overall group effect was observed for push-ups, post hoc comparisons did not reveal statistically significant differences between specific groups, suggesting that improvements in this variable were relatively consistent across intervention subgroups.

Post hoc comparisons revealed that improvements in sit-ups were observed only in the HF% group compared with Ctrl, whereas jumping jacks improved exclusively in the NF% group. In contrast, both the NF% and HF% groups showed significant improvements in mountain climbers and burpees relative to the control group, with particularly marked improvements in the HF% group for burpees. Superscript letters indicate significant differences compared with the control group (a *p* < 0.05, b *p* < 0.01, c *p* < 0.001).

Collectively, these findings suggest that baseline adiposity was associated with differences in the magnitude of physiological adaptations, particularly in metabolic outcomes. However, improvements in functional performance appeared to be less dependent on initial body composition, as both the NF% and HF% groups demonstrated consistent gains relative to the control condition.

## 4. Discussion

The present findings demonstrate that a 12-week combined training program integrating FT, HIIT, and aquatic exercise improves aerobic capacity, body composition, metabolic biomarkers, and functional performance in middle-aged and older women. Overall, the findings indicate that this multicomponent intervention is an effective strategy to induce meaningful physiological adaptations associated with improved cardiometabolic health and functional capacity. Importantly, baseline adiposity was associated with differences in the magnitude of metabolic adaptations, particularly in aerobic capacity and markers related to glucose regulation and lipid profile.

The increase in VO_2_max observed in this study suggests that the combined FT, HIIT, and aquatic-based training stimulus provides sufficient cardiovascular overload to enhance aerobic capacity, even in populations typically characterized by reduced exercise tolerance. This adaptation is likely mediated by improvements in mitochondrial function, oxygen utilization, and cardiovascular efficiency, which are well-documented physiological responses to high-intensity interval training [16]. These findings are consistent with previous studies reporting significant improvements in VO_2_max following high-intensity interval-based exercise [17,18], reinforcing the potential of time-efficient training strategies to improve cardiorespiratory fitness in middle-aged and older women [3,4,5].

In addition to aerobic adaptations, the observed increase in skeletal muscle mass indicates that the inclusion of resistance-based and functional training components effectively stimulates muscle protein synthesis and neuromuscular adaptations. This is particularly relevant in aging populations, where the preservation of muscle mass is critical to counteract sarcopenia and maintain functional independence [19]. Although no significant changes were detected in body fat percentage or BMI, these results highlight that improvements in body composition may occur through qualitative changes in tissue composition rather than reductions in total body mass, underscoring the importance of assessing muscle-related outcomes [8].

From a metabolic perspective, the reduction in fasting insulin levels and the improvement in lipid profile parameters reflect enhanced metabolic efficiency and improved insulin sensitivity. These adaptations are likely driven by increased skeletal muscle glucose uptake, improved insulin signaling pathways, and enhanced lipoprotein metabolism, including greater lipoprotein lipase activity and lipid oxidation [20,21,22].

Although HOMA-IR did not reach statistical significance, the observed trend suggests a potential improvement in insulin resistance that may become more pronounced with longer intervention durations or larger sample sizes.

The subgroup analysis revealed that baseline adiposity influenced the magnitude of certain physiological adaptations. Participants in the HF% group exhibited greater improvements in VO_2_max and fasting insulin, which suggests that individuals with higher initial metabolic impairment may experience greater relative benefits from structured exercise interventions. This response may be attributed to a greater capacity for metabolic improvement, as individuals with higher adiposity often present more pronounced baseline dysregulation [23].

Interestingly, despite differences in metabolic responses, improvements in functional performance were consistently observed across both the NF% and HF% groups. This suggests that functional adaptations to exercise may be less dependent on baseline adiposity and more closely related to neuromuscular and coordination-related mechanisms [24]. These findings reinforce the value of multicomponent training interventions in promoting functional capacity across diverse populations, regardless of body composition status.

These findings may also be interpreted within the broader context of structured exercise interventions conducted in populations with different adiposity profiles and age ranges. Previous studies in overweight and obese adolescents have demonstrated that structured physical activity programs can induce significant improvements in cardiometabolic health, aerobic fitness, and quality of life, regardless of whether interventions are primarily aerobic, resistance-based, or combined in nature [25,26]. For example, Fanelli et al. reported that structured exercise interventions in adolescents with overweight or obesity improved metabolic and functional outcomes, highlighting the relevance of individualized exercise strategies across different stages of life [25]. Similarly, the HEARTY trial demonstrated that combined aerobic and resistance training elicited favorable adaptations in obese youth, particularly regarding cardiometabolic risk reduction and functional health improvements [26].

Although the present study focused on middle-aged and older women, these previous findings support the concept that baseline adiposity may modulate the magnitude of exercise-induced adaptations across populations. Collectively, the evidence suggests that multicomponent and structured exercise interventions represent an effective strategy to improve health-related fitness and metabolic outcomes in individuals with elevated adiposity, despite differences in age and physiological characteristics.

An important aspect to consider is the discrepancy observed between statistical significance and effect size in certain variables. This highlights the importance of complementing traditional null hypothesis testing with effect size interpretation to better understand the practical relevance of training-induced adaptations, particularly in studies with relatively small sample sizes [27].

From an applied perspective, these findings support the implementation of combined FT + HIIT programs as an effective and time-efficient strategy to improve health-related fitness in middle-aged and older women [3,4,5,9]. Furthermore, the inclusion of aquatic exercise enhances the accessibility and sustainability of the intervention, making it particularly suitable for populations with physical limitations or joint-related concerns [28]. The differential response observed according to baseline adiposity also suggests that exercise prescription may benefit from a more individualized approach, particularly when targeting metabolic outcomes.

The inclusion of aquatic exercise may have contributed not only to the observed physiological adaptations but also to improved adherence and reduced joint stress, which may partially explain the consistent improvements in functional performance observed across participants.

Despite these findings, several limitations should be acknowledged. The relatively small sample size may limit the generalizability of the results. Additionally, dietary intake and physical activity outside the intervention were not strictly controlled, which may have influenced body composition and metabolic outcomes. Furthermore, aerobic capacity was estimated using a submaximal field test, which may introduce some degree of measurement variability [13]. Additionally, confidence intervals for effect sizes and change scores were not calculated, which limits the precision and interpretability of the magnitude of the observed effects. Therefore, the results should be interpreted cautiously, and future studies are encouraged to include confidence intervals to strengthen the robustness of statistical inferences. Future research should incorporate larger randomized controlled trials, longer intervention periods, and more detailed mechanistic assessments to further elucidate the interaction between baseline adiposity and responsiveness to multicomponent training interventions.

## 5. Conclusions

A 12-week multicomponent training program, including aquatic-based exercise, may improve aerobic capacity, skeletal muscle mass, fasting insulin, lipid profile, and functional performance in middle-aged and older women. These findings support multicomponent training as a feasible and effective strategy to promote health-related fitness in aging populations.

Baseline adiposity may influence the magnitude of some metabolic adaptations, with greater improvements in VO_2_max and fasting insulin observed in women with higher body fat percentage. In contrast, functional performance improved across training subgroups, suggesting that neuromuscular benefits may occur regardless of initial adiposity status.

Although body fat percentage and BMI did not change significantly, the increase in skeletal muscle mass highlights the importance of including muscle-related outcomes when evaluating exercise interventions in older women.

These findings should be interpreted with caution due to the quasi-experimental design, relatively small sample size, and absence of covariance adjustment for baseline differences. Additionally, the exploratory nature of subgroup analyses limits the ability to draw definitive conclusions regarding the role of baseline adiposity. Future randomized controlled trials are warranted to confirm these results and further clarify the role of baseline adiposity in modulating training responses.

## Figures and Tables

**Table 1:** Weekly structure of protocol combining functional training (FT) and high-intensity interval training (HIIT).

Week	Session Number	Training Type	Exercises
1	1	HIIT	Step-up, Jumping jacks, Shuttle sprint, Jab core, Fartlek
	2	FT	Quadriceps curl, Femoral curl, Hip thrust, Body weight squat
	3	HIIT/FT	Jab core, Shuttle sprint, Step-up, Hip thrust, Quadriceps curl, Femoral curl
2	1	HIIT	Rope jumping, Superman hold, Jumping jacks, Tire mountain climbers
	2	FT	Bench press, Shoulder press, Lateral raises, Triceps dips, Skullcrusher
	3	HIIT/FT	Pool noodle front float, High knees, Pool noodle lateral float, 10 m shuttle runs
3	1	HIIT	High knees, Lateral leg swim, Low skip, Front leg swim, Fartlek
	2	FT	Toes-to-bar, Biceps curl, Plank, Push-ups, Crunches
	3	HIIT/FT	High knees, Low skip, Fartlek, Toes-to-bar, Plank, Crunches
4	1	HIIT	Jab core, Tire mountain climbers, Plank jacks, Lateral shuffle, Step-up
	2	FT	Dumbbell lunges, Femoral curl, Leg press, Dumbbell flys, Rowing
	3	HIIT/FT	Kickboard drill, Pool noodle front float, 10 m shuttle runs, Pool noodle lateral float

Note: This table summarizes the session type, training modality, and exercises performed during the 12 sessions of each 4-week training cycle, which was repeated three times to complete the 12-week intervention. HIIT sessions consisted of 50 min circuit training performed in two rounds (30 s work, 20 s rest between exercises, and 50 s rest between rounds). FT consisted of three sets performed at 60% of the estimated one-repetition maximum (1RM) during weeks 1–6, with progressive load increases of 5% every two weeks, reaching 75% 1RM by week 12. As the load increased, the number of repetitions per set was reduced by two.

**Table 2:** Baseline characteristics of study participants (*n* = 34).

Variables	Ctrl (*n* = 10)	Intervention (*n* = 24)	*p*-Value
Age (years)	59.80 ± 6.19	59.00 ± 6.51	0.743
Weight (kg)	70.89 ± 20.10	74.73 ± 14.11	0.528
Body fat (%)	40.75 ± 5.12	37.50 ± 10.33	0.730
Skeletal muscle mass (kg)	22.24 ± 5.10	22.00 ± 3.91	0.884
BMI (kg/m^2^)	28.37 ± 5.34	28.17 ± 6.01	0.926
Glucose (mg/dL)	99.20 ± 7.94	98.00 ± 10.92	0.756
Insulin (μIU/mL)	12.23 ± 5.19	16.74 ± 9.01	0.139
Triglycerides (mg/dL)	134.30 ± 61.77	128.20 ± 54.95	0.662
Total cholesterol (mg/dL)	196.10 ± 45.22	185.40 ± 53.58	0.583
HDL cholesterol (mg/dL)	35.50 ± 12.40	36.67 ± 9.95	0.730

Note: All variables are expressed as mean ± standard deviation (SD).

**Table 3:** Changes in aerobic capacity, body composition, and metabolic biomarkers following the intervention (*n* = 34).

Variables	Ctrl Pre	Ctrl Post	Δ Ctrl	Intervention Pre	Intervention Post	Δ Intervention	*p*	Cohen’s d
VO_2_max (mL·kg^−1^·min^−1^)	14.15 ± 2.33	14.36 ± 2.23	0.21	12.55 ± 1.16	15.71 ± 1.06	3.16	0.035	1.10
Skeletal muscle mass (kg)	22.24 ± 1.61	22.31 ± 1.59	0.07	22.00 ± 0.79	23.05 ± 0.75	1.05	0.038	0.81
Body fat (%)	40.75 ± 1.61	39.71 ± 1.62	−1.04	37.50 ± 2.10	36.33 ± 2.07	−1.17	0.849	−0.07
BMI (kg/m^2^)	28.37 ± 1.69	28.13 ± 1.70	−0.24	28.17 ± 1.22	27.63 ± 1.23	−0.54	0.415	−0.31
Glucose (mg/dL)	99.20 ± 2.51	100.50 ± 4.24	1.30	98.00 ± 2.23	98.08 ± 3.02	0.08	0.835	−0.08
Insulin (μIU/mL)	12.23 ± 1.64	11.47 ± 1.71	−0.76	16.74 ± 1.84	12.10 ± 0.92	−4.64	0.030	−0.79
HOMA-IR	2.96 ± 0.37	2.72 ± 0.97	−0.24	4.13 ± 2.46	2.96 ± 1.29	−1.84	0.094	−0.65
Triglycerides (mg/dL)	134.30 ± 19.53	140.40 ± 22.42	6.10	128.20 ± 11.22	113.50 ± 9.49	−14.70	0.029	−0.66
Total cholesterol (mg/dL)	196.10 ± 14.30	189.70 ± 14.61	−6.40	185.40 ± 10.94	174.20 ± 9.67	−11.20	0.014	−0.22
HDL cholesterol (mg/dL)	35.50 ± 3.92	31.00 ± 2.90	−4.50	36.67 ± 2.03	44.17 ± 2.28	7.50	<0.001	1.53
TG/HDL	1.98 ± 0.70	1.74 ± 0.57	−0.24	1.47 ± 0.27	1.02 ± 0.15	−0.45	0.382	−0.28

Note: Data are presented as mean ± SEM. Δ represents change scores (post–pre). Between-group comparisons (control vs. intervention) were performed using independent sample *t*-tests or Mann–Whitney U tests, as appropriate. Statistical significance was set at *p* < 0.05. Effect sizes (Cohen’s d) were calculated based on change scores and interpreted as trivial (<0.2), small (0.2–0.49), moderate (0.5–0.79), and large (≥0.8).

**Table 4:** Changes in functional performance outcomes assessed by the McCloy physical fitness test following the 12-week intervention (*n* = 34).

Variables	Ctrl Pre	Ctrl Post	Δ Ctrl	Intervention Pre	Intervention Post	Δ Intervention	*p*	Cohen’s d
Squats	22.40 ± 1.05	23.00 ± 1.26	0.60	21.29 ± 0.86	25.67 ± 0.74	4.38	0.008	1.06
Push-ups	18.00 ± 1.14	18.90 ± 0.94	0.90	17.71 ± 0.77	21.75 ± 0.99	4.04	0.058	1.00
Sit-ups	20.70 ± 1.17	21.20 ± 1.17	0.50	21.29 ± 0.79	25.67 ± 0.64	4.38	0.005	1.12
Jumping jacks	41.60 ± 2.60	44.30 ± 1.50	2.70	37.00 ± 1.60	46.08 ± 1.72	9.08	0.006	0.98
Mountain climbers	51.40 ± 3.39	53.90 ± 2.65	2.50	44.54 ± 2.13	62.04 ± 3.28	17.50	0.005	1.13
Burpees	12.70 ± 0.78	13.40 ± 0.45	0.70	10.25 ± 0.54	16.21 ± 0.48	5.96	0.011	2.85

Note: Data are presented as mean ± SEM. Δ represents change scores (post–pre). Between-group comparisons (control vs. intervention) were performed using independent sample *t*-tests or Mann–Whitney U tests, as appropriate. Statistical significance was set at *p* < 0.05. Effect sizes (Cohen’s d) were calculated based on change scores and interpreted as trivial (<0.2), small (0.2–0.49), moderate (0.5–0.79), and large (≥0.8).

**Table 5:** Exploratory changes in aerobic capacity, body composition, and metabolic biomarkers according to baseline adiposity levels, Ctrl, normal fat (NF%) and high fat (HF%).

Variables	Ctrl Pre	Ctrl Post	Δ Ctrl	NF% Pre	NF% Post	Δ NF%	HF% Pre	HF% Post	Δ HF%	*p*
VO_2_max (mL·kg^−1^·min^−1^)	14.15 ± 2.33	14.36 ± 2.23	0.21	15.12 ± 1.09	17.13 ± 1.14	2.01	10.70 ± 1.70	14.70 ± 1.61	4.00 ^b^	0.004
Skeletal muscle mass (kg)	22.24 ± 1.61	22.31 ± 1.59	0.07	20.36 ± 0.94	21.26 ± 0.92	0.90	23.18 ± 1.11	24.33 ± 0.99	1.15	0.107
Body fat (%)	40.75 ± 1.61	39.71 ± 1.62	−1.04	26.32 ± 0.69	25.46 ± 1.14	−0.86	45.49 ± 1.21	44.09 ± 1.11	−1.40	0.780
BMI (kg/m^2^)	28.37 ± 1.69	28.13 ± 1.70	−0.24	25.43 ± 1.52	25.09 ± 1.56	−0.34	30.12 ± 1.64	29.44 ± 1.67	−0.68	0.113
Glucose (mg/dL)	99.20 ± 2.51	100.50 ± 4.24	1.30	100.50 ± 3.20	97.40 ± 1.47	−3.10	96.14 ± 3.06	98.57 ± 5.14	2.43	0.686
Insulin (μIU/mL)	12.23 ± 1.64	11.47 ± 1.71	−0.76	13.88 ± 1.60	10.70 ± 0.93	−3.18	18.79 ± 2.87	13.11 ± 1.41	−5.68 ^b^	0.018
HOMA-IR	2.96 ± 0.37	2.72 ± 0.97	−0.24	3.45 ± 0.42	2.58 ± 0.23	−0.87	4.62 ± 0.79	3.23 ± 0.41	−1.39	0.128
Triglycerides (mg/dL)	134.30 ± 19.53	140.40 ± 22.42	6.10	126.30 ± 13.12	110.60 ± 13.64	−15.70	129.50 ± 17.21	115.50 ± 13.45	−14.00	0.234
Total cholesterol (mg/dL)	196.10 ± 14.30	189.70 ± 12.77	−6.40	168.60 ± 19.14	149.10 ± 16.98	−19.50	197.40 ± 12.46	192.10 ± 9.05	−5.30	0.241
HDL cholesterol (mg/dL)	35.50 ± 3.92	31.00 ± 2.90	−4.50	34.20 ± 2.32	40.00 ± 3.00	5.80 ^a^	38.43 ± 3.04	47.14 ± 3.11	8.71 ^c^	0.001
TG/HDL	1.98 ± 0.70	1.74 ± 0.57	−0.24	1.30 ± 0.43	1.12 ± 0.33	−0.18	1.58 ± 0.35	0.95 ± 0.14	−0.63	0.256

Note: Data are presented as mean ± SEM. Δ represents change scores (post–pre). Between-group comparisons (Ctrl, NF%, and HF%) were performed using one-way ANOVA or Kruskal–Wallis tests, as appropriate. When significant differences were detected, post hoc analyses were conducted using control group as reference. Statistical significance was set at *p* < 0.05. Superscript letters indicate significant differences compared with control group: ^a^ *p* < 0.05, ^b^ *p* < 0.01, ^c^ *p* < 0.001.

**Table 6:** Changes in functional performance outcomes according to baseline adiposity levels (Ctrl, NF%, HF%) following the 12-week intervention.

Variables	Ctrl Pre	Ctrl Post	Δ Ctrl	NF% Pre	NF% Post	Δ NF%	HF% Pre	HF% Post	Δ HF%	*p*
Squats	22.40 ± 1.05	23.00 ± 1.26	0.60	21.10 ± 0.90	25.80 ± 0.74	4.70	21.43 ± 1.37	25.57 ± 1.00	4.14	0.067
Push-ups	18.00 ± 1.14	18.90 ± 0.94	0.90	18.60 ± 1.37	22.60 ± 1.90	4.00	17.07 ± 1.06	21.14 ± 1.07	4.07	0.046
Sit-ups	20.70 ± 1.17	21.20 ± 1.17	0.50	22.10 ± 0.94	26.20 ± 0.53	4.10	20.71 ± 1.19	25.29 ± 1.04	4.58 ^a^	0.021
Jumping jacks	41.60 ± 2.60	44.30 ± 1.50	2.70	37.50 ± 2.84	47.90 ± 3.01	10.40 ^a^	36.64 ± 1.94	44.79 ± 2.06	8.15	0.035
Mountain climbers	51.40 ± 3.39	53.90 ± 2.65	2.50	45.10 ± 4.55	66.40 ± 6.86	21.30 ^a^	44.14 ± 1.88	58.93 ± 2.76	14.79 ^b^	0.004
Burpees	12.70 ± 0.78	13.40 ± 0.45	0.70	12.30 ± 0.61	17.90 ± 0.67	5.60 ^b^	8.78 ± 0.56	15.00 ± 0.48	6.22 ^c^	<0.001

Note: Data are presented as mean ± SEM. Δ represents change scores (post–pre). Between-group comparisons (Ctrl, NF%, and HF%) were performed using one-way ANOVA or Kruskal–Wallis tests, as appropriate. When significant differences were detected, post hoc analyses were conducted using control group as reference. Statistical significance was set at *p* < 0.05. Superscript letters indicate significant differences compared with control group: ^a^ *p* < 0.05, ^b^ *p* < 0.01, ^c^ *p* < 0.001.

## Data Availability

The data presented in this study are available on reasonable request from the corresponding author. The data are not publicly available due to privacy and ethical restrictions related to participant confidentiality.

## Abbreviations

VO_2_max	Maximal oxygen uptake
BMI	Body mass index
FT	Functional training
HIIT	High-intensity interval training
HDL	High-density lipoprotein
HOMA-IR	Homeostatic model assessment of insulin resistance
SEM	Standard error of the mean
NF%	Normal fat percentage group
HF%	High fat percentage group
